# Overnutrition Determines LPS Regulation of Mycotoxin Induced Neurotoxicity in Neurodegenerative Diseases

**DOI:** 10.3390/ijms161226190

**Published:** 2015-12-10

**Authors:** Ian James Martins

**Affiliations:** 1 Centre of Excellence in Alzheimer’s Disease Research and Care, School of Medical Sciences, Edith Cowan University, 270 Joondalup Drive, Joondalup 6027, Australia; i.martins@ecu.edu.au; Tel.: +61-8-6304-2574; 2 School of Psychiatry and Clinical Neurosciences, The University of Western Australia, Nedlands 6009, Australia; 3 McCusker Alzheimer’s Research Foundation, Hollywood Medical Centre, 85 Monash Avenue, Suite 22, Nedlands 6009, Australia

**Keywords:** cholesterol, mycotoxin, amyloid beta, apolipoprotein E, dyslipidemia, insulin resistance, Alzheimer’s disease, lipopolysaccarides, liver, brain

## Abstract

Chronic neurodegenerative diseases are now associated with obesity and diabetes and linked to the developing and developed world. Interests in healthy diets have escalated that may prevent neurodegenerative diseases such as Parkinson’s and Alzheimer’s disease. The global metabolic syndrome involves lipoprotein abnormalities and insulin resistance and is the major disorder for induction of neurological disease. The effects of bacterial lipopolysaccharides (LPS) on dyslipidemia and NAFLD indicate that the clearance and metabolism of fungal mycotoxins are linked to hypercholesterolemia and amyloid beta oligomers. LPS and mycotoxins are associated with membrane lipid disturbances with effects on cholesterol interacting proteins, lipoprotein metabolism, and membrane apo E/amyloid beta interactions relevant to hypercholesterolemia with close connections to neurological diseases. The influence of diet on mycotoxin metabolism has accelerated with the close association between mycotoxin contamination from agricultural products such as apple juice, grains, alcohol, and coffee. Cholesterol efflux in lipoproteins and membrane cholesterol are determined by LPS with involvement of mycotoxin on amyloid beta metabolism. Nutritional interventions such as diets low in fat/carbohydrate/cholesterol have become of interest with relevance to low absorption of lipophilic LPS and mycotoxin into lipoproteins with rapid metabolism of mycotoxin to the liver with the prevention of neurodegeneration.

## 1. Introduction

Interests in lowering peripheral cholesterol levels to reduce the risk of Alzheimer’s disease (AD) have been the focus of many research studies, with particular impact for the regulation of brain amyloid beta (Aβ) metabolism that is closely connected to neurodegenerative disease. Neurodegeneration and brain cholesterol disorders have been the subject of intense research by many laboratories [1,2,3] since the peripheral metabolism of cholesterol is not linked to brain cholesterol metabolism. Cholesterol cannot pass through the blood brain barrier (BBB) with brain cholesterol homeostasis maintained by cholesterol excretion in form of oxysterols. The brain must obtain cholesterol from de novo synthesis with astrocytes, and oligodendrocytes mainly involved in cholesterol synthesis and neurons account for only a small amount of the brain cholesterol [4]. Brain cholesterol homeostasis is maintained by cholesterol excretion in the form of 24S-hydroxysterol (24S OHC), accomplished by the cytochrome P450 species, and in man the brain release approx. 6 mg of 24S OHC into the periphery each day is removed predominantly by the liver [4]. In AD patients studies have shown that cholesterol metabolism is disturbed with elevated 24S OHC levels possibly related to neuronal death and neurodegeneration [4]. An integrated approach to advance our knowledge require the identification of toxic dietary components such as bacterial lipopolysaccharides (LPS) and fungal mycotoxins that have become of importance to neuron apoptosis in neurodegenerative disease, *versus* the brain cholesterol dyshomeostasis that is linked to Aβ oligomerization, that determines neuron survival for the treatment of neurodegeneration and AD.

The interests in hypercholesterolerolemia with low high density lipoproteins (HDL) and high low density lipoproteins (LDL) in the plasma of AD patients [4] has increased with relevance to diet, nutrition and assessment of insulin resistance (obesity, diabetes)/atherosclerosis with elevated phospholipid transfer activity (PLTP) [5,6,7] associated with neurodegeneration and AD. Diets that are healthy stabilize insulin resistance, increase HDL, and promote neuron and synapse maintenance in the brain [1,4]. The consumption of mycotoxins by mycotoxin contamination in food [8,9,10,11,12,13,14,15,16] has adverse human health [17] effects and now has become of global interest. Mycotoxin contamination has been found in cereal/grains, coffee (ochratoxin A), apples, apple juice, nuts, and nut products [8,9,10,11,12,13,14,15,16,17]. Mycotoxins are the toxic secondary metabolites of molds and fungi and many of which are pathogenic to humans. Mycotoxins enter the body through the skin, digestive tract, or through respiration and when the body with aging accumulates a few milligrams [8,9,10,11,12,13,14,15,16,17] the lethal effects of mycotoxins have been associated with various organs.

**Figure 1:**
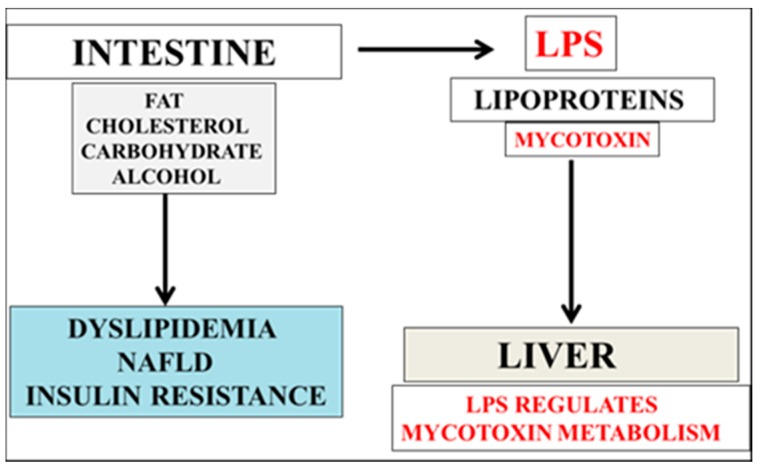
Links between diet and neurodegeneration has escalated with the involvement of bacterial lipopolysaccarides and fungal mycotoxins in amyloid beta (Aβ) homeostasis. Nutrition and food science with diets that promote the low absorption of lipophilic LPS and mycotoxin into lipoproteins with the rapid hepatic metabolism of LPS/mycotoxins to the liver, have become important to prevent early neurotoxicity and to promote the survival of neurons with age.

Diets high in cholesterol (Figure 1) may promote rapid absorption of various mycotoxins with increased membrane cholesterol that sequester mycotoxins [18]. Sequestration of mycotoxins by a cholesterol model system has now become an important technology to prevent mycotoxin toxicity [18]. Furthermore, mycotoxin levels are associated with hypercholesterolemia and have detrimental effects on liver cholesterol metabolism by effects on cholesterol synthesis [19]. Mycotoxins have been shown to bind to lipoproteins in human and animal plasma and induce hypercholesterolemia [20]. The effects of ingestion of a cholesterol diet involve intestinal transport of cholesterol in lipoproteins such as chylomicrons and chylomicron remnants with delivery of cholesterol to the liver [21,22]. The liver synthesizes very low density lipoprotein (VLDL) and transports cholesterol to peripheral tissues [23].

These lipoproteins have generally spherical structures consisting of a neutral lipid core of cholesteryl esters and triglycerides and a surface layer of phospholipids, unesterified cholesterol and various apolipoproteins such as apolipoproten E and apoliprotein AI [24]. Mycotoxins are lipophilic [25] and after ingestion of fat and cholesterol can be incorporated into lipoproteins (Figure 1) with sequestration by cholesterol in the surface of lipoprotein particles. Interests in lipoprotein sequestration may allow reduction in mycotoxin toxicity [19]. The effects of delivery of mycotoxins to the liver [25] interfere with the VLDL and HDL cholesterol metabolism and are responsible with hypercholesterolemia and atherosclerosis [19]. In various studies in animals the role of mycotoxins in neurological disease [26,27,28,29,30] have indicated effects on oxidative stress pathways involved in neurotoxicity [31]. Mycotoxins such as macrocyclic trichothecenes [32,33,34] and ochratoxin A (OTA) have been shown to induce apoptosis of neuronal cells [35,36,37] and contribute to the pathogenesis of neurodegenerative diseases such as AD and PD. Other cells such as hepatocytes, platelets (cholesterol levels), macrophages, endothelial cells, and astrocytes are susceptible to mycotoxin toxicity. Mycotoxins have an acute or chronic effect in man and depend on the nature of mycotoxin and the composition of the diet that are associated with the development of diseases of the liver (cancer), kidney, lungs, reproductive system, and gastrointestinal tract that may also involve the immune system and hormone imbalances. Interests in the effects of mycotoxin and its transport to the brain have accelerated with the current global NAFLD epidemic with corruption of mycotoxin clearance by the liver with relevance to accelerated neurodegeneration and amyloidosis.

The effects of bacterial lipopolysaccharides (LPS) on dyslipidemia indicate that the abnormal clearance and metabolism of fungal mycotoxins in lipoproteins are linked to hypercholesterolemia and neurodegeneration (Figure 1). The synergistic effect of LPS and mycotoxins interfere by post transcriptional and post translational modifications to cells and disturb cholesterol interacting proteins, lipoprotein metabolism, and membrane apo E/Aβ interactions relevant to hypercholesterolemia with close connections to Alzheimer’s disease. Hypercholesterolemia is one of the major causes of NAFLD and blood brain barrier disease (BBB) and with aging the plasma oxysterol levels increase in chronic diseases [38,39,40,41,42,43] that accelerate the transport of HDL to the brain [44]. HDL is closely linked to oxysterol induced cell damage and as oxysterol contents increase in HDL the prevention of oxysterol damage to peripheral cells and neurons occurs. In AD brain oxysterol levels are increased and disturb the BBB associated with increased Aβ production [45,46]. Hypercholesterolemia and NAFLD induce BBB disease with increased LPS and mycotoxin transport to the brain with neurodegeneration. Individuals with insulin resistance such as obese and diabetic individuals have BBB disease [47] and transport of LPS and mycotoxin corrupt BBB disease important to drug therapy associated with accelerated brain aging. The cholesterol lowering drug Simvastation has been shown to block disturbed BBB disease induced by hypercholesterolemia [48].

In AD BBB disease has been reported and its early detection has become important to the prevention of neurodegeneration [49,50]. The BBB microvasculature is composed of a capillary basement membrane containing endothelial cells, astrocyte end feet, and pericytes inside the basement membrane. Transport of mycotoxins that are non-polar cross the BBB rapidly *in vivo* [51,52] or may involve LPS induced BBB permeability [53,54] to mycotoxin transport across BBB membranes. Myoctoxins such as T-2 or HT-2 toxin cause BBB disruption by the influence on the tight junction in the BBB [55]. Mycotoxins may corrupt BBB membranes by their incorporation into the membranes that induce membrane changes and lipid peroxidation [31]. LPS induced BBB permeability has been shown and involves increased reactive BBB opening with the transport of proteins across the BBB such as insulin [54] and with the impairment of the BBB transport of Aβ from the brain [56,57] with corruption of the peripheral Aβ clearance pathway. Hypercholesterolemia induced BBB permeability that involve oxysterols are also linked to LPS and mycotoxin induction of BBB disease in NAFLD and neurodegeneration.

Diets that contain alcohol, fat, and carbohydrate stimulate the absorption of LPS [58,59,60,61] that can rapidly insert into cell membranes, with a preference for insertion and partition into cholesterol/sphingomyelin domains in lipoproteins and cell membranes [62,63] with relevance to mycotoxin transport. LPS has been shown to effect cholesterol efflux, with effects on hepatic lipoprotein metabolism and dyslipidemia [64,65,66,67,68]. LPS administration in mice are closely linked with connections to insulin resistance with effects on NAFLD, systemic inflammation and the metabolic syndrome. The importance of LPS on the regulation of cholesterol and Aβ homeostasis [69,70,71] involve apolipoprotein E (apo E-PLTP) that are closely linked to NAFLD [64,65,66,67,68] and involve the metabolism of liver mycotoxins [25,26]. The synergism of LPS and mycotoxin on NAFLD involve the corruption of peripheral Aβ metabolism [69,70,71], associated with increased plasma cholesterol, oxysterol, mycotoxin levels, and low HDL levels involved with early neurotoxicity in various neurodegenerative diseases.

## 2. Mycotoxin and LPS Regulate Cholesterol and Aβ Metabolism

The role of cholesterol in modulating the expression of amyloid precursor protein (APP) and the levels of cell Aβ have been reported [4,7] and Aβ metabolism connected to the low density lipoprotein receptor (LDLr) family [4]. Cholesterol modulates Aβ levels and Aβ acts on lipid metabolism by effects on cholesterol synthesis that may play an important role in sphingomyelin/ceramide metabolism [4]. In the brain and liver, the LDLr family play an important role in the metabolism of cholesterol and Aβ with the LDL receptor related protein 1 (LRP1) closely linked to AD [72,73,74]. LRP1 and LDLr acts on the blood brain barrier (BBB), and regulate the transport of Aβ to the periphery from the brain [72,73,74]. The corruption of LPS of peripheral and brain Aβ transport [69,70,71] involve disturbed membrane cholesterol homeostasis with membrane cholesterol involved in mycotoxin sequestration [19,25].

Cholesterol is an essential membrane component that influences hepatocyte and neuron function and in neurons, cholesterol is critical for the maintenance of synaptic connections. Membrane lipids such as cholesterol play an important role with cholesterol related proteins in neurons that conduct electrical impulses in association with membrane proteins. In membranes the association of phospholipids, glycosphingolipids such as ceramide or gangliosides, and glycerophospholipids (plasmalogen) with cholesterol have marked effects on membrane protein structure and function with the regulation of ion pumps [4,7]. Mycotoxins such as patulin in cultured cells have been shown to interfere with membrane function and ion channels involved in the transduction of extracellular an intracellular signals [25,75]. Interactions with membrane cholesterol involve mycotoxin sequestration and LPS that bind to the cholesterol/sphingomyelin domains in membranes [62,63].

LPS are endotoxins and essential components of the outer membrane of all Gram-negative bacteria. Bacterial LPSs are dimeric molecules consisting of a polysaccharide moiety linked to a lipid core termed Lipid A which is anchored within the cell membrane. Lipid rafts/caveolae containing sphingomyelin and cholesterol form microdomains in cell membranes for the recruitment of lipid modified proteins such as Aβ oligomers. LPS may influence membrane cholesterol by binding to cell membranes and lipoproteins and its packing in the membrane allows the increased interaction or displacement of the Aβ peptide, that leads to electrostatic Aβ oligomer formation and fibril formation [69].

LPS disturbance in cell cholesterol efflux involve the liver X receptor and ATP-binding cassette transporter proteins (LXR-ABCA1) interactions [76]. Furthermore, LPS interference with cholesterol interacting proteins such as caveolin-1, which is a major component of caveolae (membrane microdomains, 50–100 nm), involve corruption of cellular cholesterol endocytosis [77,78,79,80,81,82,83,84,85,86,87,88]. LPS has been shown to effect caveolin expression [89,90], apo AI [91,92,93] and apo E [69] with effects on neutralization of cholesterol efflux that involve ABCA1 [94,95,96]. The effects of LPS on plasma cholesterol metabolism involve HDL metabolism with neutralization of apo AI [91,92,93] associated with low HDL levels. Increased membrane cholesterol levels induced by LPS delay hepatic mycotoxin metabolism in membranes. Increased cholesterol levels in membranes have been associated with increased Aβ oligomers with toxic effects on membrane lipid peroxidation [69,70,71,97] that may involve cholesterol interacting proteins such as the GPCRs [98,99,100,101,102,103]. LPS effects on caveolin-1 expression involve ABCA1 and insulin receptor levels with effects on Aβ production and relevance to AD [104,105,106,107,108,109,110,111].

## 3. LPS/Mycotoxin Interactions Interfere with Apolipoprotein E/Aβ Peptide Interactions and Determine Neuron Survival

Novel information on dietary components that perturb the interactions between two key peptides (apo E and Aβ) has the potential to considerably improve brain (neuron) and liver Aβ metabolism with relevance to insulin resistance and neurodegeneration [112]. The understanding of specific toxins such as bacterial LPS and mycotoxins that interfere with the transport of apo E mediating Aβ clearance in the brain and liver is clearly needed. The integrated approach will advance our knowledge of the importance of diet [4] in this transport process relevant to cholesterol/Aβ homeostasis, that provide insights in relation to the toxic process that leads to neurodegeneration and AD.

Apo E is an important apolipoprotein in lipid metabolism with multiple roles in cell biology [112]. Interactions of apo E isoforms with the Aβ peptide have been studied in various laboratories [112] to understand how apo E4 promotes risk for neurodegeneration. The hypothesis that the interaction between these peptides is determined by the nature of associated lipids such as cholesterol which alters their conformation and determines the cellular uptake of the apo E/Aβ complex is important to the field of AD. The role of the apo E (E2, E3, E4) isoforms and their specific interactions with the Aβ peptide have been reported and integrates cholesterol metabolism with AD [112].

**Figure 2:**
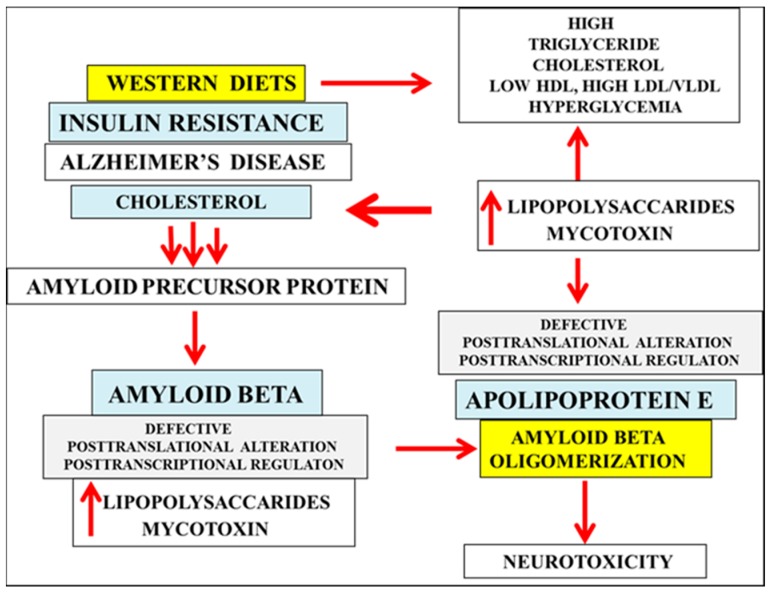
Bacterial LPS and mycotoxin promote dyslipidemia, electrostatic Aβ oligomer formation via post translational or post transcriptional modifications. (1) Bacterial LPS and mycotoxin induce dyslipidemia and NAFLD; (2) LPS and mycotoxin alter cell and membrane cholesterol homeostasis with increased Aβ formation; (3) Defective Sirtuin 1 (Sirt 1) post transcriptional regulation and post-translational modifications of cholesterol interacting proteins by LPS/mycotoxin neutralize apo E and increase Aβ oligomerization with connections to insulin resistance and AD; (4) Electrostatic Aβ oligomer formation may be independent of the early neuron apoptosis associated with neurotoxicity in individuals with insulin resistance and neurodegenerative diseases (Parkinson’s disease and Alzheimer’s disease).

The kinetics of binding between apo E and Aβ are determined by different isoforms of apo E and LPS has now been shown to neutralize apo E-PLTP activity [69,70,71] with the corruption of the apo E-Aβ interaction [69,70,71,112]. Apo E4 and its association with protein misfolding of Aβ has been studied extensively and cell membrane LPS and mycotoxin contents have become important to apo E3 individuals with relevance to susceptibility to Aβ oligomer formation and neurodegeneration (Figure 2). Membrane-bound and soluble proteins have been shown to bind LPS such as LPS binding protein (lipoproteins), toll-like receptor (TLR), GPCR, and CD14 receptor. In the central nervous system, systemic LPS injection initiates the acute phase response and upregulates membrane CD14 receptor that controls TLR4 endocytosis [59,113] and induces microglial activation that results in neurodegeneration and Parkinson’s disease (PD) [114]. The CD14 receptor is referred to as the LPS receptor and is involved in the phagocytosis of the Aβ peptide [115,116,117,118]. Mycotoxins have also been shown to be involved in CD14 receptor expression in macrophages [119]. LPS induction of APPs [69,70] are linked to the CD14 receptor with the levels linked to liver inflammation and NAFLD. LPS and the mycotoxin patulin have been shown to effect hepatic genomic stability [69] with effects on reverse cholesterol transport in macrophages and with macrophage activation that involve LPS and mycotoxin [59,113,119].

LPS in brain cells improves the scientific understanding of membrane cholesterol and Aβ oligomer formation in the brain. Astrocytes have been shown to accumulate neuronal Aβ to prevent Aβ plaque development [120,121,122,123]. In the brain the HDL are the major lipoproteins involved in the turnover of brain cholesterol [124]. The astrocyte synthesizes apo E-PLTP with apo E-PLTP that play an important role in cholesterol and Aβ metabolism [125,126,127,128] that determine neuron synapse formation. The disturbances in brain HDL (apo AI) lipoprotein metabolism induced by LPS [91,92,93] also effect astrocyte apo E [69] and the LDLr [129] with increased brain cholesterol linked to poor neuron survival with connections to electrostatic Aβ oligomer formation [130]. Furthermore, LPS corruption of the apo E and hepatic Aβ metabolism are associated with disturbed brain cholesterol levels relevant to disturbed apo E-PLTP activity involved in the regulation of Aβ [130]. Furthermore, LPS regulated cholesterol sequestration of mycotoxins in cell membranes displace Aβ peptides with abnormal effects of mycotoxin such as patulin on various biological functions that regulate membrane fluidity and ion channel function in cells from various tissues [25,131,132,133,134,135].

LPS and mycotoxins regulate acute phase proteins such as serum amyloid protein A [69,112,136] and C reactive protein [137,138] involved in amyloidosis in AD. LPS has been shown to reduce the release of albumin from the liver in man with relevance to the principal role of albumin in peripheral and brain Aβ aggregation [70]. In rats, patulin had marked effects on liver albumin and the plasma protein fraction that was found to be markedly decreased was albumin [139]. Therefore the peripheral effects of LPS and patulin nullify the albumin mediated Aβ and apo E mediated transport from the brain [70].

## 4. Patulin and LPS Effect Electrostatic Aβ Oligomer Formation via Post Translational or Post Transcriptional Modifications

Specific amino acids such as cysteine are involved in post-translational modifications that include oxidation, nitrosylation, or disulfide bond formation [140,141] with electrostatics that have and play important roles in biology such as toxic Aβ oligomer formation. Cysteine residues are important to many protein functions and patulin has been shown to induce cysteine intra and inter molecular crosslinks that effect many cholesterol interacting proteins such as GPCR receptor activation, apo AI-ABCA1 cholesterol dependent efflux, LRP-1 associated LPS/mycotoxin metabolism, LDLr binding of lipoproteins, apo E (E2, E3, E4)/Aβ interactions, and phospholipid transport protein synthesis/secretion that effects apo E-PLTP activity [138,142,143,144,145,146,147,148,149,150,151,152]. The mycotoxin patulin has been shown to be neurotoxic and in mice patulin has been shown to induce brain damage [153,154]. Patulin has been shown to induce intra- and intermolecular protein crosslinks *in vitro* of other amino acids such as lysine, histidine side chains, and alpha-amino groups [155]. These three amino acids (cysteine, lysine, histidine) are found in Aβ [156,157,158,159,160] with effects of patulin on these amino acids that possibly are responsible for Aβ electrostatic nature and promotion of Aβ oligomer formation.

Interest in dietary regulation of liver LPS and mycotoxin metabolism involves Sirtuin 1 (Sirt1). Sirt 1 is one of the nuclear receptors known to regulate several cell functions by deacetylating both histone and non-histone targets [161,162]. Sirt 1 is a NAD(+)dependent class III histone deacetylase protein that targets transcription factors to adapt gene expression to metabolic activity, insulin resistance and inflammation in chronic diseases [163,164]. Nutritional regulation (calorie restriction and high fat feeding) of Sirt1 is involved in neuron proliferation with effects on cellular cholesterol closely linked to Aβ clearance in AD [165,166,167]. In mammalian cells, patulin has been shown to have toxic effects in the nucleus [153,154] with posttranslational modification of cysteine residues by patulin relevant to the nutrient sensing nuclear receptor Sirt 1 that contains a zinc centre (activator) co-ordinated by four cysteine residues that are critical for its function [168]. Many mycotoxins such as patulin interfere with DNA repair in liver cells [169] and override Sirt 1’s involvement in DNA repair and telomere maintenance in cells [170]. Patulin and its toxic effects on cysteine cross links in Sirt 1 dysfunction may be relevant to the corruption of nitric oxide and neural pathways [171]. LPS and mycotoxins are involved with disturbed cellular nitric oxide homeostasis with relevance to nitric oxide dyshomeostasis and Aβ metabolism [171]. Nitric oxide neurotoxicity [171,172] induced by LPS and mycotoxins may generate the strong oxidant peroxynitrite [173,174] involved in the inhibition of Sirt 1 binding to zinc [168,175].

Sirt 1 is involved in the regulation of nuclear liver X receptors linked to ABCA1 (cholesterol metabolism) targets in the liver and brain [4]. The interest in neuron glucose metabolism has accelerated with the role of Sirt 1 and its role in transcriptional regulation of p53 [176,177] linked to p53 transcriptional regulation of caveolin 1 expression [178,179,180] associated with insulin receptor transport and activity [105,106,107,108]. LPS has been shown to repress Sirt 1 [177,181] and caveolin expression [89,90] with implications to class B, type I scavenger receptor [67] involved in cholesterol trafficking and HDL levels. Sirt 1 downregulation in the liver and brain prevents mycotoxin metabolism with effects on NAFLD and neurodegeneration. Injections with l-cysteine prevented the effect of LPS in liver injury [182] with apo AI and HDL linked to LPS neutralization [91,92,93].

Synergism of LPS and mycotoxin interactions in cells involving accelerated inflammation and patulin levels should be monitored to prevent complete neuron apoptosis and brain Aβ oligomerization with implications to shifts in protein homeostasis (Figure 2). The effects of LPS on inflammation in the BBB have been reported to involve caveolin-1 and α-synuclein [183,184,185,186]. LPS and relevance to Aβ/α-synuclein transport and insulin resistance has escalated [71] with relevance to BBB permeability and corruption of the neuronal Aβ transport across the BBB to the periphery for metabolism by the liver. The effects of LPS on the release of acute phase proteins [69,70,112] involve interactions with Aβ and α-synuclein oligomers to delay or promote Aβ oligomerization.

LPS and α-synuclein involve corruption of membrane cholesterol flux to HDL and mycotoxin transport from peripheral cells/neurons by binding of α-synuclein to membrane cholesterol (isooctyl chain) to prevent cholesterol metabolism [187]. The LPS repression of Sirt 1 [181] promotes defective cholesterol efflux in the periphery and brain with increased levels of alpha synuclein and Aβ (Figure 2). α-synuclein effects on increased BBB permeability induced by LPS/α-synuclein interactions [69,70,71] promote the transport of mycotoxins into the brain and in the absence of LPS the BBB transport of mycotoxins may not be relevant. In the aging process, the increased concentrations of LPS are transported to the brain (LPS mediated) and its neurotoxic effect on apelin-Sirt 1 interactions induce alterations in NO homeostasis [167] and determine neuron survival [188]. Furthermore, patulin [155] may have direct toxic effects on the inhibition of the apo E and Aβ cysteine interaction in membranes and determine Aβ oligomer formation and NO toxicity [189,190] in the brain with early aging and AD. Furthermore, electrostatic Aβ oligomer formation may involve α-synuclein (cysteine, histidine) by the effects of patulin on intra and intermolecular protein crosslinks involving cysteine, lysine, and histidine side chains [155] involved in protein oligomerization [71,191,192]. Patulin effects on lipid-protein interactions may also involve disturbed crosslinks that involve Aβ histidine 13 binding to ganglioside [193] associated with Aβ aggregation.

## 5. Nutritional Diets Reduce Neurotoxins and Allow Effective Drug Treatment Programs

LPS dysregulation of nuclear Sirt 1 [177,181] has become important with relevance to hepatic cholesterol metabolism [76,77,78] that are now closely connected to cellular mycotoxin (ng–µg) and Aβ metabolism [71]. As aging occurs Sirt 1 is dysregulated [194,195,196] in the developing world with Sirt 1 downregulation now associated with various chronic diseases. Diets that are healthy do not contain LPS [197,198] or mycotoxin [10,11,15,25] with both components involved with disturbed cell cholesterol homeostasis/Aβ metabolism and absence of these components from the human plasma allow effective drug therapy such as statin drug treatment that may maintain proper synaptic function and neuron survival. Statins, patulin, and Aβ have been shown to inhibit protein prenylation [199,200,201] with effects on cell cholesterol sequestration and Aβ oligomer formation.

Food patulin levels should be reduced with relevance to the toxic effects of patulin [202] in peripheral tissues. Patulin levels [203] have been measured and daily intake can be high and found in foods such as fresh and dried fruits, processing stages of bread, apple (apple juice concentrates 50 µg/kg), and cereals. Patulin levels in apple juice concentrates, blue cheese, and cereals have been clearly documented. Bacterial lipopolysaccharides are also found in unpasteurized fruit juices, ciders, bread (oven baked poorly), and fruits (diverse bacterial populations) [197,198] and toxic effects of patulin may involve LPS interactions. Strict surveillance of apple ripening and cheese making/ripening has to be implemented to ensure patulin levels are low with increased levels associated with the aging process.

Consumption of apples and cheese are a rich source of pyruvic acid (amino acid metabolism) and leucine and ingestion of these nutrients has been associated with activation of Sirt 1 [195]. The potent effects of apple and cheese spoilage that produces patulin is concentrated in the liver with the induction of NAFLD in the developing world. The OTA that is found in the rind and inner part of the cheese and in alcohol (beer, wine) rise in the blood plasma with NAFLD linked to increased OTA transport to the brain. Alcohol (apple cocktail, beer, wine) promotes the intestinal absorption of LPS/patulin/OTA and alcohol is a Sirt 1 inhibitor [170]. Alcohol contains patulin/OTA [204] with relevance to the hepatic metabolism of mycotoxin and Aβ.

The failure of the anti-obese drug program in Western countries [205] with central acting CNS drugs may be better understood with high fibre diets that activate Sirt 1 in the liver with the maintenance of hepatic fatty, cholesterol, glucose metabolism that is closely connected to hepatic LPS/mycotoxin metabolism. The antipsychotic/antidepressants used to treat neurodegeneration interact with cell membranes [181] and the role of patulin and LPS interactions (nitric oxide toxicity) may be involved with ineffective drug therapy in the brain. High fibre diets that contain both fruits and vegetables (phystosterols) improve Aβ metabolism [4] but mycotoxin/LPS contamination [10,11,15,25,197,198] disrupt membrane phytosterol therapy with LPS/mycotoxin contamination associated with the packing, storage, transportation, handling, and processing of fresh produce to the final destination. Phytosterol consumption regulates hepatic cholesterol metabolism with the liver involved with rapid detoxification (hepatic biotranformation enzymes, cytochrome p450) of mycotoxins by elimination into bile in animals and man [206].

In developing countries, the increase in xenobiotics [207] in polluted environments such as drugs, drug metabolites, synthetic pesticides, and herbicides may delay mycotoxin metabolism with mycotoxin related neurotoxicity. Diets that activate the Sirt 1/pregnane X receptor pathway respond by the expression of cytochrome p450 that may allow rapid metabolism of xenobiotics (food, air, water) and mycotoxins (food, air, water) [207] with reduced neurotoxin transport to the brain. The xenobiotics consumed may corrupt the hepatic elimination of mycotoxins (*vice versa*) with the induction of NAFLD that is now closely linked to neurodegeneration.

Interests in increased hepatic cholesterol metabolism and transport to the bile has increased with phosphatidylinositol (PI) doses [208] essential for lowering liver cholesterol with increased plasma apo AI (HDL levels). The cell membrane carries a net negative surface charge with the presence of anionic lipids, which constitute 10% of the total lipids in the plasma membrane [112]. PIs in cell membranes consist of 1% of the lipids that are physiopathological modulators of membrane cholesterol and amyloid homeostasis [112]. LPS and patulin effects on cell membranes corrupt PI and Aβ membrane interactions [112]. The addition of PI (2–5 gm/meal) accelerated liver cholesterol metabolism over a two-week period [208] with relevance of PI ingestion to improve hepatic LPS/mycotoxin metabolism. Mycotoxins/LPS are closely connected with platelet function and appropriate PI consumption is required to assist with platelet cholesterol levels, Aβ production, and prevention of platelet aggregation [32,33,209,210,211,212]. The importance of consumption of nuts as a rich source of PI has accelerated with relevance to doses of PI ingestion that override caveolin-1 downregulation (Sirt 1) that is a phosphatidylinositol 3-kinase sensitive [213] with the prevention of hypercholesterolemia, NAFLD, and amyloidosis [7,176,177,194,195,196]. However, nut consumption should be carefully monitored with relevance to NO composition and mycotoxin content [15].

## 6. Conclusions

Food and nutrition guidelines for the handling and processing of fresh fruit, bread, and vegetables are essential and fresh produce may require cold preservation procedures to prevent minimal bacterial and fungi contamination of food. In the developing world, the food content of LPS/mycotoxin levels determine nuclear receptor defects such as Sirt 1 downregulation involved in the survival of neurons. Small amounts of LPS/mycotoxin in food/alcohol that are absorbed into the plasma, are rapidly cleared by the liver when liver Sirt 1 is under circadian regulation. The diabetes epidemic (Sirt 1 senescence) in the developing world may related to the appetite dysregulation, with absorption of higher contents of LPS/mycotoxins that induce early insulin resistance and cholesterol efflux disturbances associated with NAFLD and neuron dysfunction relevant to various chronic diseases. Diets that are Western style (high calorie, cholesterol) may promote increased absorption of xenobiotics that induce NAFLD and neurodegeneration. Drugs and xenobiotics consumed (drug regime) by various obese and diabetic individuals should be assessed since common pathways shared with xenobiotics/LPS/mycotoxins may not allow hepatic metabolism of LPS/mycotoxins (dyslipidemia) with increased delivery of ochratoxin A (coffee), patulin (apple), and LPS (cheese, fruit, vegetables) to the brain, associated with early neurodegeneration and independent of the structural conversion to electrostatic Aβ oligomer formation.
